# SPISE INDEX (Single point insulin sensitivity estimator): indicator of insulin resistance in children and adolescents with overweight and obesity

**DOI:** 10.3389/fendo.2024.1439901

**Published:** 2024-11-22

**Authors:** Giacomo Tantari, Marta Bassi, Angela Pistorio, Nicola Minuto, Flavia Napoli, Gianluca Piccolo, Alberto La Valle, Giordano Spacco, Carla Cervello, Giuseppe D’Annunzio, Mohamad Maghnie

**Affiliations:** ^1^ Pediatric Clinic and Endocrinology Unit, Istituto di Ricovero e Cura a Carattere Scientifico (IRCCS) Istituto Giannina Gaslini, Genoa, Italy; ^2^ DINOGMI (Department of Neuroscience, Rehabilitation, Ophthalmology, Genetics, Maternal and Child Health), University of Genoa, Genoa, Italy; ^3^ Epidemiology and Biostatistics Unit, Scientific Directorate, IRCCS Istituto Giannina Gaslini, Genoa, Italy; ^4^ Neuro-Oncology Unit, IRCCS Istituto Giannina Gaslini, Genova, Italy

**Keywords:** obesity, overweight, insulin resistance, type 2 diabetes mellitus, adolescence

## Abstract

**Background:**

Insulin resistance in children and adolescents with obesity is linked to increased risk of type 2 diabetes mellitus and cardiovascular disease. The SPISE index, based on values of fasting triglycerides (mg/dL), HDL cholesterol (mg/dL), and BMI (kg/m2), shows promise in predicting insulin resistance in children.

**Methods:**

This study aimed to identify a SPISE cut-off for detecting insulin resistance and evaluate its relationship with pubertal development, anthropometrics, and glycometabolic profile in 232 children and adolescents, 105 males and 127 females (median age 13.2 years) with overweight (n=48) and obesity (n=184). SPISE index was calculated with the formula: 600 x HDL Cholesterol^0,185^/Triglycerides^0,2^x BMI^1,338^, and patients were categorized based on Tanner stages [(Group 1 (18.8%) Tanner 1, Group 2 (44.6%) Tanner 2-3-4, Group 3 (36.6%) Tanner 5)].

**Results:**

A SPISE cut-off ≤ 6.92 or ≤ 6.13 (based on the method used for insulin resistance detection), in subjects with Tanner stages I and II, showed good sensitivity and specificity as a marker of insulin resistance. SPISE index decreased significantly with the advancement of pubertal status (P < 0.0001) and with worsening severity of obesity (P < 0.0001). While no significant differences in SPISE marker were observed between patients with normal and abnormal glucose tolerance during OGTT within any pubertal stage, SPISE values were significantly lower in patients with confirmed insulin resistance (total sum of insulin OGTT ≥ 535 µu/mL) in all three pubertal groups (Group 1: P=0.008; Group 2: P=0.0008 and Group 3: P=0.002, respectively).

**Conclusions:**

In children and adolescents with obesity the SPISE index can be proposed as an alternative to OGTT and other insulin-based methods for evaluating insulin resistance. Its advantage lies in using readily available and inexpensive laboratory tests, making it suitable for large-scale studies and follow-up monitoring across diverse populations.

## 1 Introduction

Obesity is a complex multifactorial and severe disease characterized by an excess of body fat due to an overtime unbalanced energy expenditure (1). During the past decades, prevalence of childhood obesity has increased worldwide, especially in low and middle-income countries (2). After a misperception, in 1998 the National Institute of Health defined obesity as a chronic disease (3).

It has been reported that 20% of children aged 2-19 years are affected by obesity, and this rate is supposed to increase 130% over the next two decades (4). In 2020 the World Health Organization (WHO) reported that 12% of children aged 7-9 years living in 33 European countries can be defined with obesity, while 39 million of children aged up to 5 years are affected by obesity worldwide (5). Overweight and obesity are recognized as the fifth cause of death worldwide, responsible of 3.4 million of deaths annually (6).

Obesity is the result of the interaction between genetic predisposition, physiologic, socioeconomic and environmental factors (7). Several causative contributors for pediatric obesity have been studied: early nutritional and epigenetic mechanisms, the thrifty epigenotype, maternal malnutrition, social contagion and gut microbiota assessment (8–10).

Besides pathogenesis, overweight and obesity starting in infancy and childhood persist in adolescence and adulthood and more than 60% of prepubertal children with overweight maintain the condition overtime. Early-onset comorbidities, i.e. Insulin Resistance (IR) and Type 2 Diabetes Mellitus (T2DM), increase the risk for cardiovascular and cerebrovascular morbidity and mortality (11, 12). Moreover, obesity predisposes to Non Alcoholic Fatty Liver Disease (NAFLD), particularly dangerous in young adolescents since its progression to non-alcoholic steatohepatitis, cirrhosis and liver cancer (13).

Since adolescence, overweight and obesity are characterized by impaired metabolic status, including insulin resistance (IR), different degrees of dysglycemia, (i.e. fasting hyperglycemia and impaired glucose tolerance), and abnormal lipid profile (14).

Among children with obesity the frequency of IR varies from 33.2 to 52.1%, due to different diagnostic methods and their cut off values (15). Beside obesity, well-known pathogenetic factors for IR are genetic predisposition (16), gestational diabetes (17), born small for gestational age (18), early postnatal weight gain (19), premature birth (20) and smoking during pregnancy (21).

Quantitative assessment is not regularly performed in routine clinical practice, despite several methods availability (22). Euglycemic/hyperinsulinemic clamp represents the gold standard for IR recognition (23), an invasive and time-consuming procedure, not applicable on routine medical practice, especially in pediatric age group.

Glycometabolic assessment can be evaluated by Oral Glucose Tolerance Test (OGTT) (24). Glucose levels at +120’ define normal glucose tolerance, impaired glucose tolerance and diabetes mellitus. Recently, in adolescents with obesity, glucose level higher than 155 mg/dl at + 60’ after glucose load is considered a risk factor for T2DM (25). Noteworthy, total insulin sum (TIS) obtained during all the times of the test is useful to define IR (26). In children and adolescents with obesity, a TIS ≥ 535 microU/ml showed the highest sensitivity/specificity for T2DM risk (27).

Moreover, several indexes based on fasting blood glucose and insulin levels have been suggested (28). In particular, Homeostatic Model Assessment (HOMA) of IR (HOMA-IR), β-cell activity (HOMA-β) and insulin sensitivity (QUICKI), all based on glucose and insulin fasting samples, are the easiest and most reproducible. On the other hand insulin secretion is pulsatile (29) and has a short half–life (30) and standardized assays are lacking (31). IR indexes should consider age, pubertal stage and gender, as different results are reported according to these parameters (32).

In alternative, non-insulin-derived indirect indexes of IR like Triglycerides/HDL-Cholesterol ratio (TG/HDL-C) have been proposed (33), with the main advantage of universal availability of serum lipid measurement. However, this method has several limitations, due to great ethnic variability in the cut off points (34).

A new marker of IR, based on a mathematical model including fasting triglycerides and HDL-cholesterol plus BMI values, named Single Point Insulin Sensitivity Estimator (SPISE) has been proposed. SPISE has a better predictive value of IR as compared to HOMA-IR and QUICKI indexes (35). Similarly, SPISE index is a useful tool for detecting abnormal glucose metabolism in overweight and obese children (36).

The primary aim of our study was to determine the values of SPISE index in a group of children and adolescents with overweight/obesity. Secondary aims were to establish the relationship between SPISE index and glycometabolic profile and its predictive value as compared to other known insulin resistance indexes.

## 2 Materials and methods

### 2.1 Patients

We evaluated SPISE index and other biochemical/glycometabolic parameters in 232 children and adolescents with overweight and obesity (105 m and 127 f) median age 13.2 years (range 10.8-15.4 years), followed in the outpatient clinic, Endocrinology and Diabetes Unit, Department of Pediatrics, Giannina Gaslini Institute, Genoa, Italy, between 2016 and 2020. Inclusion criteria were: overweight or with obesity, according to WHO criteria (37), age range 10-18 years, Caucasian and Hispanic origin, availability of an OGTT. Exclusion criteria were: type 1 diabetes mellitus (T1DM), syndromic/genetic obesity, acute illnesses, administration of drugs affecting glucose metabolism., African-American origin.

### 2.2 Study design

In our cross-sectional single-center retrospective study all patients’ height, weight, body mass index, and pubertal stage according to Tanner were recorded. Measurements were performed with the subject wearing only light indoor clothing and no shoes. Height was measured with a portable Harpenden stadiometer by Tanner technique. Weight was measured with a standardized portable scale (Seca 704 ^®^). BMI was calculated as follows: weight in Kg/(height in meters)^2^. According to the WHO criteria (37), overweight was defined as BMI > 1 SDS and obesity as BMI > 2 SDS. Severe obesity was defined as BMI-for-age above + 3 Z-scores.

BMI was calculated and BMI SDS score (BMI-SDS) was computed for each subject by using the formula: BMI-SDS = (actual BMI – mean BMI for age and sex)/BMI SD for age, race, and gender, based on established standards and norms.

Pubertal development stages were assessed using Tanner staging criteria by well-trained physicians in pediatric endocrinology. Patients were divided according to the pubertal development as follows: Group 1: Tanner Stage 1; Group 2: Tanner Stages 2-3-4; Group 3: Tanner Stage 5.

SPISE index was calculated according to the formula: 600 x HDL^0.185^/Triglycerides^0.2^ x BMI^1.338^, with fasting HDL cholesterol and Triglycerides expressed in mg/dL and BMI as kg/m2 (35).

Triglicerydes and total, HDL and LDL cholesterol were detected using enzymatic colorimetric methods (38, 39).

After 8-12 hours of overnight fasting, all subjects underwent baseline diagnostic blood sample withdrawals including fasting Plasma Glucose (PG), HbA1c, insulin, triglycerides, total and HDL cholesterol levels. Glucose was detected by the glucose oxidase method on venous whole blood, and results were modified into plasma glucose values. Insulin was measured with an ElectroChemiLuminescence Immuno Assay (ECLIA) (40). All parameters were measured at the same Laboratory.

After the load, glucose tolerance was defined using standard parameters, i.e.:

- Normal Glucose Tolerance (NGT) = PG < 140 mg/dl at 2-h of OGTT,
- Impaired Glucose Tolerance (IGT) = PG 140-200 mg/dl,
- and Diabetes Mellitus (DM) = PG ≥ 200 mg/dl (41).

As estimates of insulin sensitivity we measured HOMA-IR using the following formula: [fasting plasma insulin in microU/ml ´ Fasting Plasma Glucose (FPG) in mmol/l]/22.5].

The study was conducted in accordance with the Declaration of Helsinki. In view of the retrospective nature of the study all the procedures were part of the routine care. Informed consent was obtained from all subjects involved in the study or their caregivers.

### 2.3 Statistical analysis

Descriptive statistics were performed; categorical variables were reported in terms of absolute frequencies and percentages; quantitative variables were reported in terms of median values and first and third quartiles (1st–3rd q).

Body Mass Index (BMI) was calculated as the ratio of body weight (kg) to squared height (meters). BMI was standardized by the LMS method (42), with gender and age adjustments, and was expressed as z-score, using the WHO tables as standard reference (43).

Comparison of frequencies was done utilizing the Chi-square test or Fisher’s exact test (in case of expected frequencies < 5).

Comparison of quantitative variables (example: SPISE index) in 2 different categories of patients (example: patients with normal glucose tolerance vs patients with impaired glucose tolerance/type 2 diabetes) was made by the Mann-Whitney U test.

Comparison of quantitative variables (example: SPISE index) in more than 2 (three or four) different categories of patients (example: patients with overweight vs patients with obesity vs patients with severe obesity) was made by the non-parametric Analysis of Variance (Kruskal-Wallis W test).

Correlation between quantitative parameters (e.g., HOMA-IR vs Total Insulin after OGTT) has been evaluated by means of Spearman’s Rank order correlation coefficient (rS). The correlation coefficient was considered as follows: rS < |0.4| weak, ≥ |0.4| to |0.59| moderate, ≥ |0.6| to |0.79| strong, and ≥ |0.8| very strong, according to Swinscow TVD (1997) (44).

ROC curve analysis (45) has been used to find the best cut-off values for the SPISE index that was postulated as a possible predictor of abnormal glucose metabolism.

All the statistical tests were two-sided and a P value < 0.05 was considered statistically significant. “Statistica” (release 9.1, StatSoft Corporation, Tulsa, OK, USA) and “Stata” (release 17.0, College Station, TX, USA) were used for all the univariate and bivariate analyses; the software MedCalc was used for the ROC curve analysis.

## 3 Results

Clinical data of the enrolled patients (n = 232) are reported in **Table 1**. Patients of both genders (female 54,7% males 45,3%) with a median age at evaluation of 13.2 years were included in the study. Only patients with overweight (20,7%), obesity (47,4%), and severe obesity (31,9%) were included in this cohort (**Table 1**).

**Table 1:** Characteristics of the study patients [N = 232].

	N. (%)
**Sex: Male**	105 (45.3%)
**Females**	127 (54.7%)
**Age at visit (years); *median (1^st^ – 3^rd^ q)***	*13.2 (10.8 - 15.4)*
**BMI SDS, *median (1^st^ – 3^rd^ q)***	*2.7 (2.2 - 3.1)*
** Overweight**	48 (20.7%)
** Obesity**	110 (47.4%)
** Severe obesity**	74 (31.9%)
Pubertal stage [N=224]:	
** Tanner: 1**	42 (18.8%)
** Tanner: 2-3-4**	100 (44.6%)
** Tanner: 5**	82 (36.6%)
Insulin resistance [N=229]:	
** Yes (TIS ≥ 300 microU/ml)**	197 (86%)
** No**	32 (14%)
Insulin resistance [N=229]:	
** Yes (TIS ≥ 535 microU/ml)**	129 (56.3%)
** No**	100 (43.7%)
Glucose tolerance [N=230]:	
** Normal**	152 (66.1%)
** Impaired glucose tolerance**	76 (33.0%)
** Type 2 Diabetes Mellitus**	2 (0.9%)

BMI, Body Mass Index; TIS, Total Insulin Sum during OGTT.

In our case series we compared SPISE values by stratifying categories based on BMI (overweight, obesity and severe obesity) (**Table 2**). Regarding pubertal development, patients were divided into 3 groups: prepubertal (Tanner Stage 1, 18,8%), undergoing puberty (Tanner Stages 2-3-4, 44,6%), and complete pubertal development (Tanner Stage 5, 36,6%) (**Table 1**).

**Table 2:** SPISE index values in different categories of patients stratified by pubertal stage and degree of obesity.

	Tanner stage: I		Tanner stages: II-III-IV		Tanner stage: V		P^#^
	No.	Median (1^st^– 3^rd^ q)	No.	Median (1^st^– 3^rd^ q)	No.	Median (1^st^– 3^rd^ q)	
Overweight (> 1 SDS)	7	9.8 (8.9 - 10.4)	17	7.5 (7.1 - 8.6)	18	5.8 (5 - 6.7)	< 0.0001
Obesity (> 2 SDS)	14	7.2 (6.9 - 7.5)	53	5.7 (5.3 - 6.4)	38	5.1 (4.7 - 5.5)	< 0.0001
Severe obesity (> 3 SDS)	21	5.3 (4.7 - 6.4)	28	4.4 (3.9 - 5)	23	3.6 (3.2 - 3.9)	< 0.0001
	P^#^	< 0.0001		< 0.0001		< 0.0001	

P^#^: Kruskal-Wallis W test (non-parametric Analysis of Variance).

IR as indicated by a TIS ≥ 300 microU/ml or ≥ 535 microU/ml was observed in 86% and 56,3% of patients, respectively. NGT was reported in 66,1%, IGT in 33%, T2DM in 0,9% (**Table 1**).

**Figure 1:**
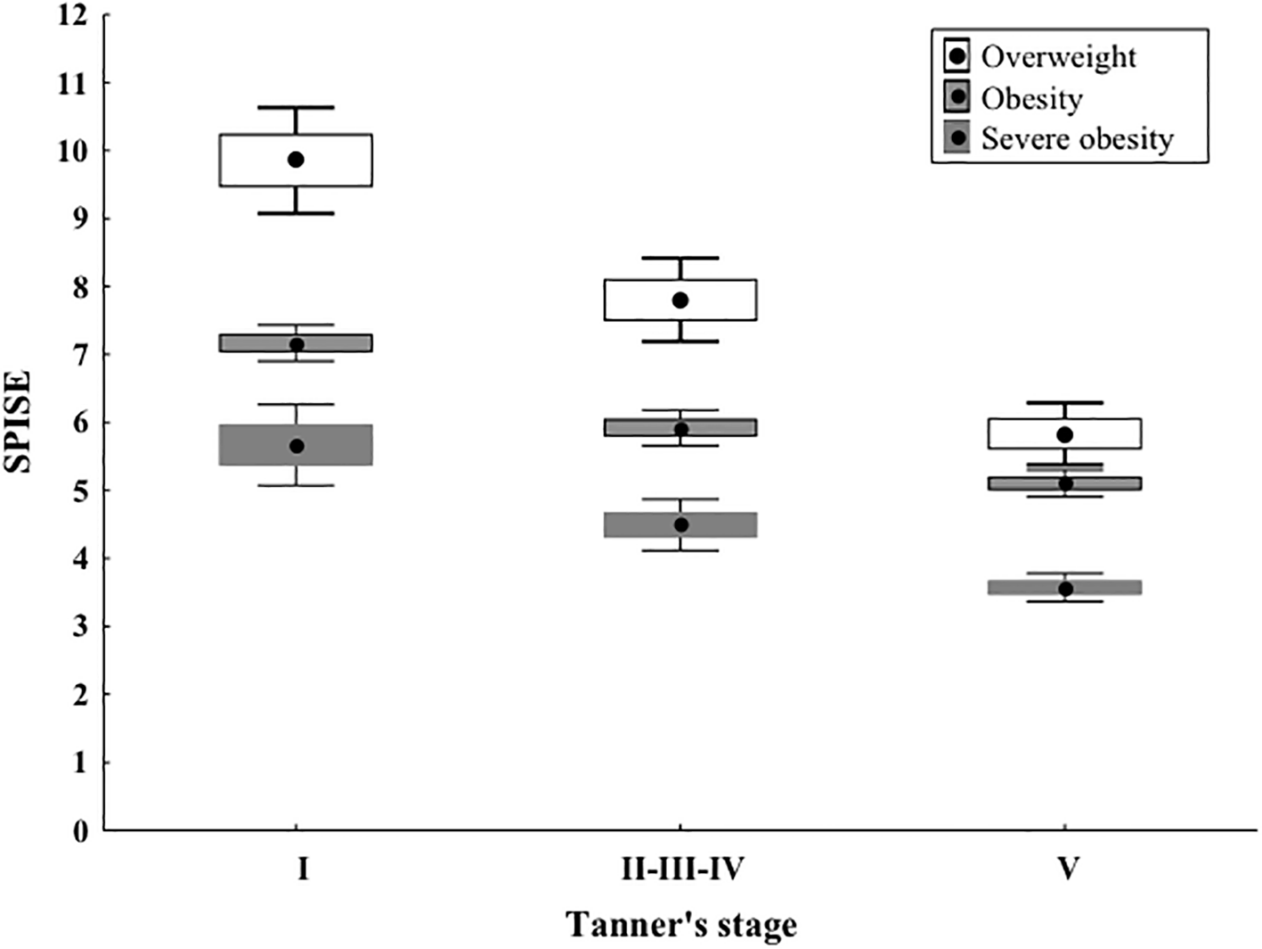
As shown in Image, the SPISE index shows a statistically significant decrease from Tanner stage I to II-III-IV and to V, in all the weight categories. Median values and quartiles as well as P values are presented in **Table 2**. In Image, differently from the table, means and 95% Confidence Intervals are presented, but the statistical test as well as the corresponding interpretation should refer to the previous table.

The SPISE index significantly decreased from Tanner 1 to Tanner 5 in all weight categories (p value < 0.0001) (**Figure 1**). The comparison of the SPISE values between prepubertal patients and those undergoing puberty with normal OGTT or impaired glucose tolerance or T2DM did not show a significant difference between the 2 groups studied (Tanner Stage 1: p value 0.66; Tanner Stages 2-3-4: p value 0.64; Tanner Stage 5 p value 0.95) (**Table 3**).

**Table 3:** SPISE index values in different categories of patients stratified by pubertal stage and glucometabolic control.

	Tanner stage: I		Tanner stage: II-III-IV		Tanner stage: V		P^#^
	No.	Median (1^st^– 3^rd^ q)	No.	Median (1^st^– 3^rd^ q)	No.	Median (1^st^– 3^rd^ q)	
NGT	28	6.7 (5.9 - 7.9)	63	5.6 (4.9 - 6.9)	54	4.7 (4.1 - 5.4)	< 0.0001
IGT/T2DM^§^	14	6.7 (5.2 - 7.4)	35	5.3 (4.9 - 6.6)	25	4.8 (3.9 - 5.7)	0.005
	P**^##^**	0.66		0.64		0.95	

**^#^** P: Kruskal-Wallis W test (non-parametric Analysis of Variance); **^##^** P: Mann-Whitney U test; IGT, Impaired glucose tolerance; NGT, Normal glucose tolerance; T2DM, Type 2 Diabetes Mellitus; ^§^only 2 patients suffered from T2DM and therefore were included in the group of patients with glucose intolerance.

Based on our previous reported findings that a total sum of insulin ≥ 535 microU/ml during OGTT had a high specificity in identifying insulin-resistance (27), we compared the SPISE values, maintaining the division into pubertal stages, in patients classified as insulin resistant (i.e. a sum of insulin at five points OGTT ≥ 535 microU/ml, compared to patients with a sum of insulin <535 microU/ml). In this case, the SPISE value was significantly lower in the group of insulin-resistant patients compared to the others, in all 3 Tanner groups (Tanner Stage 1: p value 0.008; Tanner Stages 2-3-4: p value 0.0008; Tanner Stage 5: p value 0.002) (**Table 4**).

**Table 4:** SPISE index values in different categories of patients stratified by pubertal stage and total insulin sum produced during oral glucose tolerance test (categorised according to different cut-off values: ≥ 535 microU/ml or ≥ 300 microU/ml).

	Tanner stage: I		Tanner stage: II-III-IV		Tanner stage: V		P^#^
	No.	Median (1^st^– 3^rd^ q)	No.	Median (1^st^– 3^rd^ q)	No.	Median (1^st^– 3^rd^ q)	
TIS < 535 microU/ml	24	7.4 (6.2 - 8.9)	37	6.4 (5.4 - 7.3)	34	5.2 (4.7 - 5.7)	< 0.0001
TIS ≥ 535 microU/ml	17	6.1 (4.7 - 6.9)	61	5.3 (4.6 - 6.2)	45	4.5 (3.6 - 5.2)	0.0001
	P^##^	0.008		0.0008		0.002	
TIS < 300 microU/ml	8	9.5 (6.4 - 10.1)	11	7.3 (6.4 – 8.0)	12	5 (4.2 - 5.8)	0.0025
TIS ≥ 300 microU/ml	33	6.5 (5.2 - 7.3)	87	5.4 (4.8 - 6.5)	67	4.8 (3.9 - 5.5)	< 0.0001
	P^##^	0.032		0.004		0.68	

^#^P: Kruskal-Wallis W test (non-parametric Analysis of Variance); ^##^P: Mann-Whitney U test. TIS, Total Insulin Sum.

Similarly, we stratified insulin-resistant patients using as a cut-off a sum of insulin at OGTT ≥ 300 microU/ml. In this case, the SPISE index was significantly lower in prepubertal patients and in patients undergoing puberty, while did not show a significant difference in the Tanner 5 patient group (Tanner Stage 1: p value 0.34; Tanner Stages 2-3-4: p value 0.004; Tanner Stage 5: p value 0.68) (**Table 4**).

Another widely used index of insulin resistance is the HOMA-IR [fasting plasma insulin in microU/ml x fasting plasma glucose (FPG) in mmol/l)/22.5]; we defined percentiles in a cohort of normal children and adolescents, and considered patients with HOMA-IR > 75^th^ percentile or > 99.2^th^ percentile as potentially being at higher risk of insulin resistance (46).

We compared the values of the SPISE index, using both the 75^th^ percentile of HOMA-IR (**Table 5**) and the 99.2^th^ percentile (**Table 5**) as insulin resistance cut-off; in both cases we obtained a significantly lower SPISE value in patients categorized as insulin resistant, in all Tanner groups (HOMA-IR > 75^th^ percentile; Tanner Stage 1: p value 0.017; Tanner Stages 2-3-4: p value 0.010; Tanner Stage 5: p value < 0.0001) (HOMA-IR > 99.2^th^ percentile; Tanner Stage 1: p value 0.08; Tanner Stages 2-3-4: p value < 0.0001; Tanner Stage 5: p value 0.008).

**Table 5:** SPISE index values in different categories of patients stratified by pubertal stage and HOMA-IR index (categorised according to different cut-off values: > 75^th^ percentile *versus* ≤ 75^th^ percentile or ≤ 99.2^nd^ percentile *versus* > 99.2^nd^ percentile).

	Tanner stage: I		Tanner stage: II-III-IV		Tanner stage: V		P^#^
	No.	Median (1^st^– 3^rd^ q)	No.	Median (1^st^– 3^rd^ q)	No.	Median (1^st^– 3^rd^ q)	
TIS < 300 microU/ml	8	9.5 (6.4 - 10.1)	11	7.3 (6.4 - 8)	12	5 (4.2 - 5.8)	0.0025
TIS ≥ 300 microU/ml	33	6.5 (5.2 - 7.3)	87	5.4 (4.8 - 6.5)	67	4.8 (3.9 - 5.5)	< 0.0001
	P^##^	0.034		0.004		0.68	
HOMA-IR ≤ 99.2^nd^ percentile	35	6.92 (5.74 - 8.47)	71	6.1 (5.1 - 7.07)	53	5.03 (4.49 - 5.69)	0.0001
HOMA-IR > 99.2^nd^ percentile	7	5.65 (4.33 - 7.18)	27	4.86 (4.14 - 5.46)	26	4.5 (3.53 - 5.17)	0.028
	P^##^	0.08		< 0.0001		0.008	

^#^P: Kruskal-Wallis W test (non-parametric Analysis of Variance); ^##^P: Mann-Whitney U test. TIS, Total Insulin Sum.

In order to define a significant cut-off of SPISE in our cohort, we created ROC curves, trying to identify significant areas under the curve. For this analysis we modified the division of patients based on Tanner, creating only 2 groups, one including Tanner Stages 1 and 2 and the other including Tanner Stages 3-4-5, due to the limited sample of prepubertal patient (Tanner 1).

**Figure 2:**
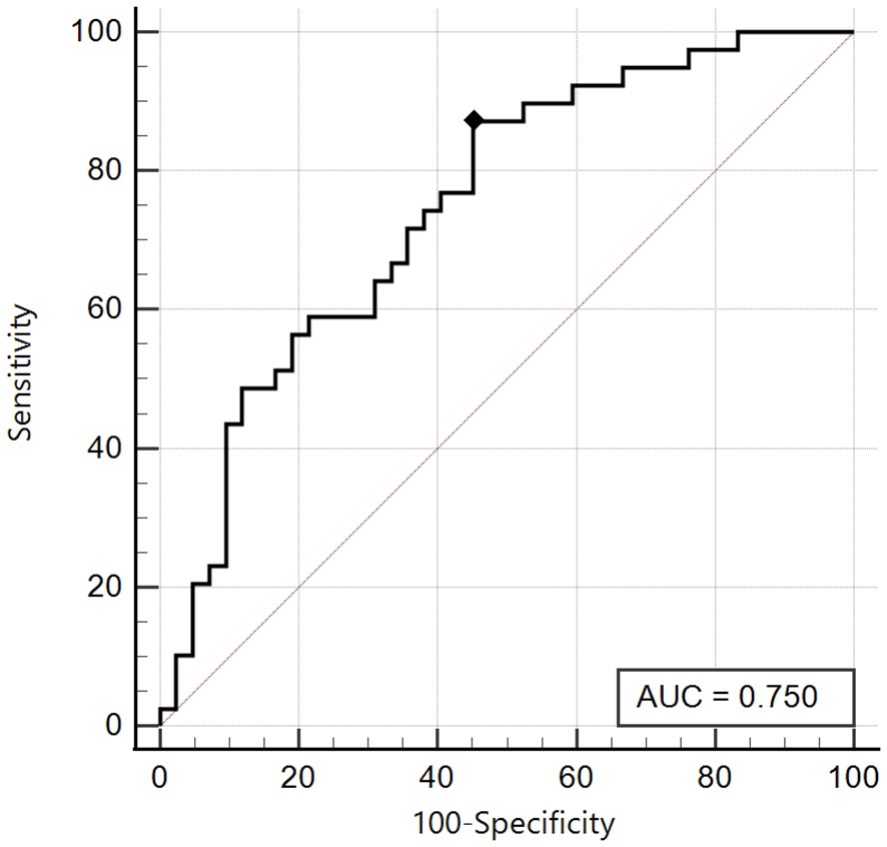
ROC curve of the SPISE Index against the categorized variable “Total Insulin Sum” (TIS) ≥ 535 microU/ml, in Tanner I-II patients [n = 39/81; 48.1%].As shown in Image, the ROC curve of SPISE against the categorised variable “Total Insulin Sum” (TIS) after OGTT ≥ 535 microU/mL, has a good value of Area Under Curve (AUC), being equal to 0.75 (95% CI: 0.64 – 0.84). The best cut-off value for SPISE in Tannerstage I and II patients, was ≤ 6.92 corresponding to a sensitivity of 87.2% and a specificity of 54.8%.

We initially compared SPISE values with patients who showed a total insulin sum on OGTT ≥ 535 microU/ml; we obtained a significant area under the curve (AUC 0.75, 95% CI: 0.64 - 0.84) in the group of patients with Tanner Stages 1-2 and the best cut-off was ≤ 6.92 with a sensitivity of 87.2% and a specificity of 54.8% (**Figure 2**).

**Figure 3:**
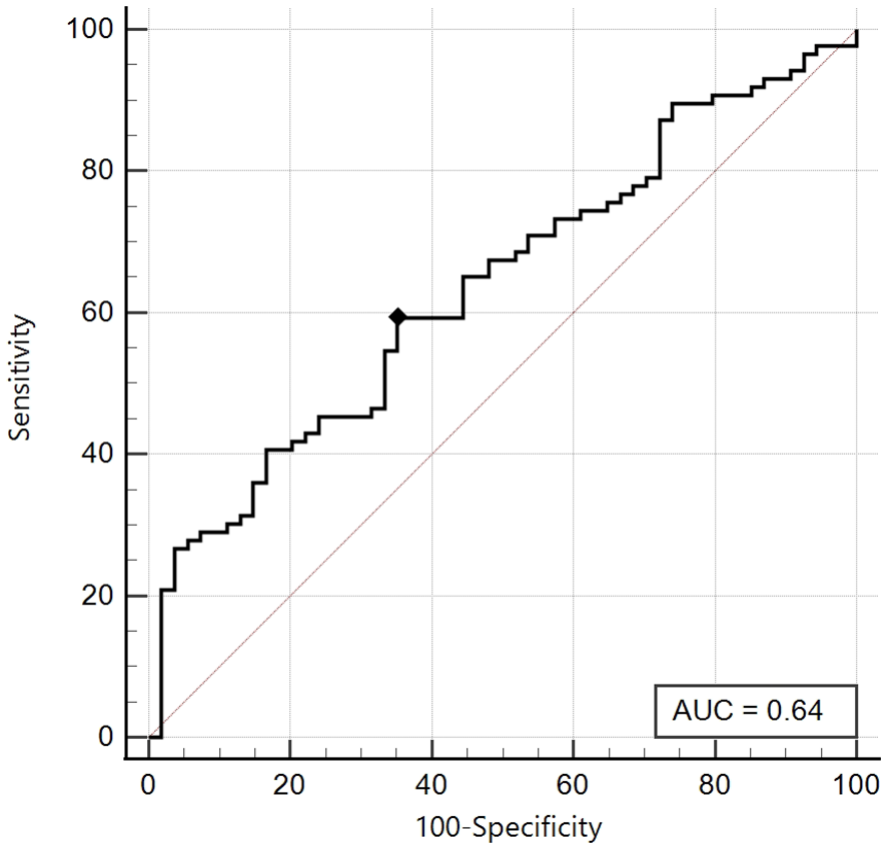
ROC curve of the SPISE Index against the categorised variable “Total Insulin Sum” (TIS) ≥ 535 microU/ml, in Tanner III-IV-V patients [n = 86/140; 61.4%].As shown in Image, the ROC curve of SPISE against the categorised variable “Total Insulin Sum” (TIS) after OGTT ≥ 535 microU/mL, has an unsatisfactory value of Area Under Curve (AUC), being equal to 0.64 (95% CI: 0.55 – 0.72). The best cut-off value for SPISE in Tannerstage III-IV and V patients, was ≤ 5.08 corresponding to a sensitivity of 59.3% and a slightly better specificity of 64.8%.

On the other hand, in the group of patients with Tanner Stages 3-4-5, the best cut-off was ≤ 5.08 with a sensitivity of 59.3% and a specificity of 64.8%, with a non-significant area under the curve (AUC 0.64, 95% CI: 0.55-0.72) (**Figure 3**).

Similarly, we created a ROC curve, using a HOMA-IR ≥ 99.2^th^ percentile as an insulin resistance parameter, maintaining the same division on the pubertal stage.

**Figure 4:**
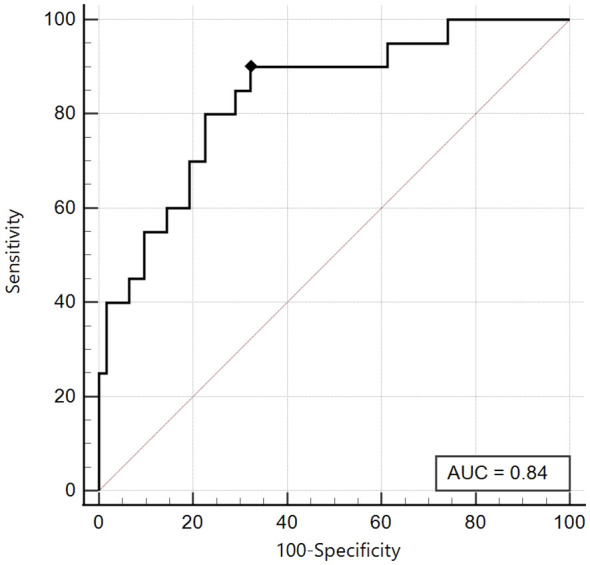
ROC curve of the SPISE Index against the categorised variable “HOMA-IR >99^th^ percentile”, in Tanner I-II patients [N = 82].As shown in Image, the ROC curve of SPISE against the categorised variable “HOMA-IR > 99^th^ percentile”, has very good value of Area Under Curve (AUC), being equal to 0.84 (95% CI: 0.74 – 0.91). The best cut-off value for SPISE in Tannerstage I-II patients, was ≤6.13 corresponding to a very good sensitivity of 90% and a specificity of 67.7%.Patients with HOMA-IR > 99^th^ percentile, in Tanner’s stage I-II, were 20 over 82, representing a percentage of24.4%].

As previously described, also in this case, we obtained for the Tanner Stages 1-2 group an excellent area under the curve (AUC 0.84, 95% CI: 0.74 - 0.91), and the best cut-off was ≤ 6.13, with an excellent sensitivity of 90% and good specificity of 67.7% (**Figure 4**).

**Figure 5:**
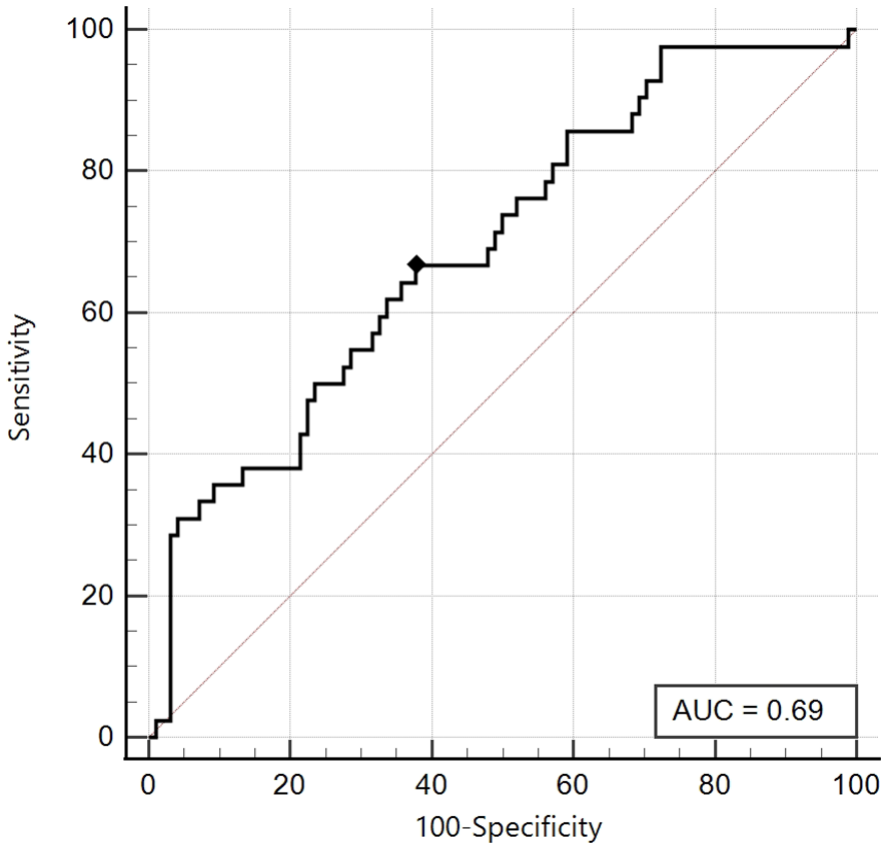
ROC curve of the SPISE Index against the categorised variable “HOMA-IR > 99^th^ percentile”, in Tanner III-IV-V patients [N = 140]. As shown in Image, the ROC curve of SPISE against the categorised variable “HOMA-IR > 99^th^ percentile”, has a only sufficient value of Area Under Curve (AUC), being equal to 0.69 (95% CI: 0.61 – 0.77). The best cut-off value for SPISE in Tannerstage III-IV and V patients, was ≤ 5 corresponding to a sensitivity of 66.7% and a slightly better specificity of 62.2%.

On the other hand, as already highlighted in the first ROC analysis, for the Tanner Stages 3-4-5 group, the area under the curve proved to be just satisfactory (AUC 0.69, 95% CI: 0.61 - 0.77) and the best cut-off was ≤ 5, with sensitivity 66.7% and specificity 62.2% (**Figure 5**).

## 4 Discussion

In our cross sectional retrospective study, we aimed at identifying a SPISE cut-off in children and adolescents with overweight and obesity in different pubertal stages. SPISE values ≤ 6.92 or ≤ 6.13, depending on the method used to determine insulin resistance [TIS ≥ 535 microU/mL (27) or HOMA-IR > 99.2^th^ percentile (46) respectively], exhibit excellent sensitivity and good specificity for identifying patients at a higher risk of IR, making it potentially applicable in clinical practice.

Our results are in agreement with Paulmichl et al., who modified the TG/HDL-C ratio, a marker of insulin resistance, and defined the SPISE index including BMI, fasting triglycerides and HDL cholesterol (35). When comparing the SPISE to other indices of insulin resistance using the area under the ROC curve (aROC) and X^2^ test, they established a cut off value of 6.61, with a better aROC than the TG/HDL-C ratio.

Our results revealed that the SPISE index decreases significantly with both increasing weight and advancing pubertal status. This observation is in line with established knowledge that insulin sensitivity decreases as children enter puberty due to physiological increases in the growth hormone/IGF-1 axis and gonadal steroids (47, 48). In particular, Correa-Burrows who evaluated the SPISE index in 725 out of 850 children and adolescents with obesity and different degrees of pubertal development found a SPISE value of 6.3 in prepubertal children with a higher sensitivity and specificity, while in pubertal patients a SPISE value of 5.4 showed the highest sensitivity and specificity for screening insulin resistance (47).

Regarding the predictive role of SPISE in patients with Tanner Stages 1 and 2, the best cut-off value of SPISE < 6.92 showed the best sensitivity and specificity, while in patients with Tanner Stages 3-4-5 the best cut-off value of SPISE was < 5.08, with lower sensitivity and specificity. In our patients with pubertal Tanner Stage 5 the SPISE value was even lower than that observed in patients undergoing puberty, as already observed by Murdock (49). As far as regards gender-based values, we did not observe any significant difference between males and females (data not shown) compared to the report by Correa-Burrows et al. of a better predictive value of SPISE as a marker of insulin resistance in males than in females (47); the lower number of our case series (232 vs 850) might explain the lack of significant difference.

In our study, the assessment of glucose tolerance during the OGTT was not significantly correlated with the SPISE index, both in prepubertal and pubertal subjects. This observation could be ascribed to the relatively young age of the study population, which is associated with a lower prevalence of impaired glucose tolerance and type 2 diabetes mellitus compared to adult or late adolescent populations studied in previous research (36, 50, 51). The analysis of SPISE and other risk factors for T2DM including a positive family history of T2DM of 104 Korean adolescents with obesity was found to significantly influence SPISE values, whose cut-off was 4.49 and significantly lower in those with T2DM as compared to normoglycemic adolescents (52).

It is worth to emphasize the role of SPISE index as a predictor of impaired glucose tolerance later in life as reported by Barchetta et al. (36) who evaluated the SPISE index in adolescents and adults with overweight/obesity. The analysis of 909 children and adolescents with overweight/obesity and 99 healthy controls showed a significantly lower SPISE index in those with impaired glucose tolerance, a positive correlation with insulin sensitivity indices and a negative relationship with age, blood pressure, HOMA-IR, basal and + 120^th^ glucose levels during OGTT, suggesting that SPISE is associated with metabolic impairment and can be considered a predictor of future glucose abnormalities (36). In our series the relatively smaller number of cases (232 vs 909) might explain the lack of difference.

Our study demonstrated promising results for the SPISE index as an indicator of IR, compared to other commonly used methods (HOMA-IR, QUICKI, Insulin during OGTT) (46). Patients classified as insulin-resistant exhibited significantly lower SPISE values, supporting its potential usefulness as a predictor of insulin resistance (35). Furthermore, the SPISE index may have an additional role in the definition of Metabolic Syndrome (MetS) although various definitions with different prevalence data exist (53). In our cohort, the lack of waist circumference and systolic blood pressure data prevented this analysis.

One of the strengths of this study is that it has identified a promising SPISE cut-off for prepubertal and Tanner 2 pubertal stage in children evaluated and followed in a single center by using rigorous clinical and biochemical parameters collected in different age groups, minimizing potential biases. Furthermore, the findings support the potential validity of the SPISE index for evaluating insulin resistance. Main limitations of this study are the relatively small sample size and the lack of a follow-up. Therefore, the study was unable to establish a reliable SPISE cut-off for later pubertal stages which requires further research with larger cohorts.

SPISE index is a promising predictor of insulin resistance, as it requires only a single fasting blood sample and could be a possible tool for large-scale population screening to identify individuals at higher risk of cardiovascular disease. Further research is needed to establish normal standardized cut-off values applicable across different age, gender and Tanner stage groups. Moreover, SPISE measurement in several illnesses characterized by insulin resistance and increased cardiovascular risk could identify those subjects requiring prevention strategies for future diseases and strict follow-up.

## Data Availability

The original contributions presented in the study are included in the article/supplementary material, further inquiries can be directed to the corresponding author/s.
